# Caveolin-1 scaffolding domain peptides enhance anti-inflammatory effect of heme oxygenase-1 through interrupting its interact with caveolin-1

**DOI:** 10.18632/oncotarget.16676

**Published:** 2017-03-29

**Authors:** Ping Weng, Xiao-Tong Zhang, Qiong Sheng, Wen-Fang Tian, Jun-Liang Chen, Jia-Jia Yuan, Ji-Ru Zhang, Qing-Feng Pang

**Affiliations:** ^1^ Wuxi Medical School, Jiangnan University, Wuxi, China; ^2^ Department of Anesthesiology, the Affiliated Hospital of Jiangnan University, Wuxi, China

**Keywords:** acute lung injury, lipopolysaccharide, heme oxygenase-1, caveolin-1, caveolin-1 scaffolding domain peptide

## Abstract

Caveolin-1(Cav-1) scaffolding domain (CSD) peptides compete with the plasma membrane Cav-1, inhibit the interaction of the proteins and Cav-1, and re-store the functions of Cav-1 binding proteins. Heme oxygenase-1 (HO-1) binds to Cav-1 and its enzymatic activity was inhibited. In this study, we investigated the effect of CSD peptides on interaction between HO-1 and Cav-1, and on the HO-1 activity *in vitro and in vivo*. Our data showed that CSD peptides decreased the compartmentalization of HO-1 and Cav-1, and increased the HO-1 activity both in LPS-treated alveolar macrophages and in mice. Meanwhile, CSD peptides obviously ameliorated the pathology changes in mice and lowered the following injury indexes: the wet/dry ratio of lung tissues, total cell numbers in bronchoalveolar lavage fluid and lactate dehydrogenase activity in the serum. Mechanistically, it was firstly found that CSD peptides promoted alveolar macrophages polarization to M2 phenotype and inhibited the IκB degeneration. Furthermore, CSD peptides down-regulated the expression of IL-1β, IL-6, TNF-α, MCP-1, and iNOS in alveolar macrophages and in lung tissue. However, the protective role of CSD peptides on LPS-induced acute lung injury in mice could be abolished by zinc protoporphyrin IX (ZnPP, a HO-1 activity inhibitor). In summary, CSD peptides have beneficial anti-inflammatory effects by restoring the HO-1 activity suppressed by Cav-1 on plasma membrane.

## INTRODUCTION

Although many modern medical technologies have been employed to treat patients with sepsis-induced acute lung injury (ALI), the mortality still remains high at 43% [1]. The major cause of high mortality is that there are currently no effective anti-inflammatory drugs for ALI [2].

Heme oxygenase-1(HO-1) degrades the free heme into carbon monoxide, iron, and biliverdin, and attenuates inflammatory response in many organs, including the lung [3]. Accumulating evidence suggests that HO-1 itself and its catalytic reaction products have important protective role in many lung disease models, including LPS-induced ALI, ischemia-reperfusion-induced lung injury [4]. However, as far as HO-1 enzymic catalytic activity is concerned, a unique event must be taken into consideration, that is, plasma membrane caveolin-1 (Cav-1) binds HO-1 and inhibit the enzymatic activity of HO-1 [5–7].

Cav-1 and HO-1 are often co-located and particularly abundant in pulmonary epithelial cells, alveolar macrophages [8, 9]. The scaffolding domain of Cav-1 (82-101 amino acid residues) inhibits the function of Cav-1 binding proteins [10–14]. Interestingly, Cav-1 plays complex roles in the pathogenesis of ALI. Cav-1 promoted polymorphonuclear adhesion, chemotaxis, and epithelial and endothelial cell apoptosis/senescence. However, Cav-1 had anti-fibrotic effect in the fibrotic phase of lung injury [8]. To date, whether this compound function of Cav-1 in ALI is involved in regulating the activity of HO-1 is yet unknown.

The common methods to deprive of the inhibitory effect of Cav-1 include siRNA or gene knockout. Compared with the two measures, Cav-1 scaffolding domain (CSD) peptides have unique advantages. When connected with Antennapedia homeodomain (AP), CSD peptides quickly entered into cells and tissues [15, 16]. Moreover, CSD peptides have particularly useful because they were functional when delivered *in vivo*. Intratracheal administration the CSD peptides blocked the progression of bleomycin-induced lung fibrosis in mice [17, 18]. AP-CSD peptides suppressed tissue edema and vascular leakage through anti-inflammatory property [19]. From all the above research, we can suppose that AP-CSD peptides may be a valuable tool for investigating the mechanisms involved in the interaction of caveolin-1 and its binded proteins *in vitro* and *in vivo*.

In this study, we hypothesized that CSD peptides could competitively interact with HO-1 and specifically disrupt the inhibitory actions of plasma membrane Cav-1 toward HO-1, and thereby enhance HO-1 bioactivity. As expected, we firstly demonstrated that CSD peptides increased HO-1 activity and alleviated LPS-induced pulmonary inflammation *in vitro and in vivo*.

## RESULTS

### Effect of the CSD peptides on co-localization of HO-1/Cav-1 and HO-1 activity in LPS-stimulated AMs

**Figure 1:**
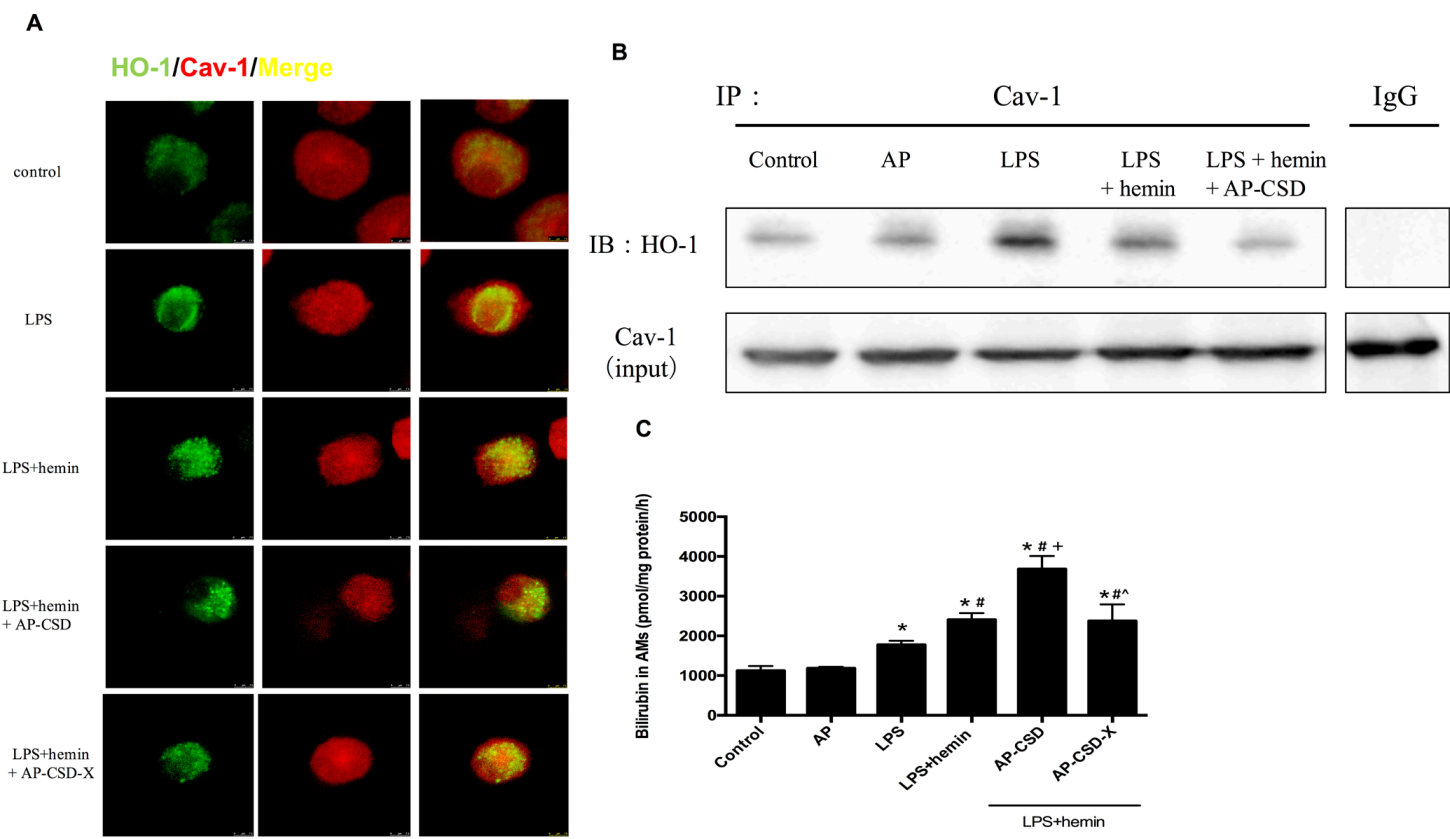
CSD peptides decreased interaction between HO-1 and Cav-1, and induced the HO-1 activity in alveolar macrophages Primary alveolar macrophages were pretreated for 6 h with or without CSD peptides, followed by LPS and hemin treatment for 16 h. **A**. The cells were immunostained with anti-HO-1 (green) and anti-Cav-1 (red) and analyzed by confocal microscopy. The merged images were shown. Scale bar = 7.5 μm. **B**. Cell lysates from alveolar macrophages were immunoprecipitated with anti-Cav-1 and immunoblotted with anti-HO-1. Total Cav-1 in cell lysates served as the loading control. **C**. HO-1 activity was determined in AMs. Values represented mean ± SD (*n* = 5 for each group). *, significance compared with control group (*P < 0.05*); #, significance compared with LPS group (*P < 0.05*); +, significance compared with LPS + hemin group (*P < 0.05*); ^, significance compared with LPS + hemin + AP-CSD group (*P < 0.05*).

In the LPS-challenged AMs, compartmentalizations of HO-1 and Cav-1 on plasma membrane were determined by confocal microscopy (Figure 1A) and co-immunoprecipitation (Figure 1B). There were significantly increased interaction between HO-1 and Cav-1 in LPS group and LPS+hemin group whereas CSD peptides could decrease the level of interaction between HO-1 and Cav-1. Meanwhile, CSD peptides added HO-1 activity compared with LPS + hemin group (Figure 1C).

### Effect of CSD peptides on inflammatory cytokine production in LPS-stimulated AMs

**Figure 2:**
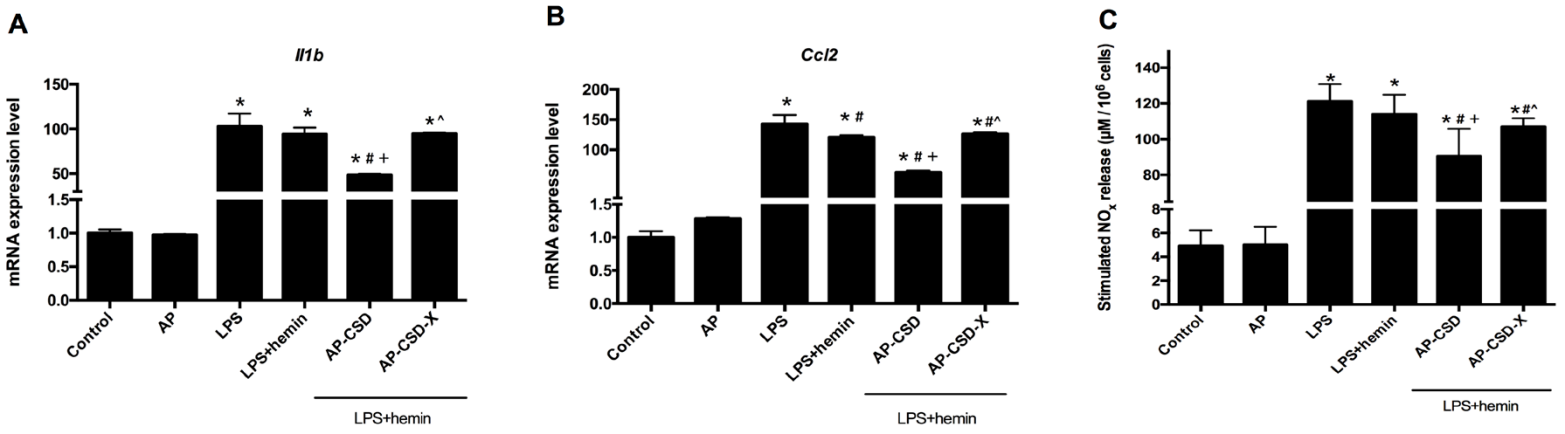
The anti-inflammatory effect of CSD peptides on LPS-stimulated alveolar macrophages **A**.-**B**. The mRNA level of *Il1b* and *Ccl2* in alveolar macrophages were determined by qPCR. Incubation with medium alone was referred as the control group. Expression in the control group was normalized to 1. **C**. The NO_x_ in culture medium was measured directly using Griess reaction. Values represented mean ± SD (*n* = 5 for each group). *, significance compared with control group (*P < 0.05*); #, significance compared with LPS group (*P < 0.05*); +, significance compared with LPS + hemin group (*P < 0.05*); ^, significance compared with LPS + hemin + AP-CSD group (*P < 0.05*).

AMs stimulated with LPS resulted in the increased expression of *Il1b* and *Ccl2* at mRNA levels. Conversely, CSD peptides could markedly suppress *Il1b* and *Ccl2* expression compared with LPS + hemin group (Figure 2A and 2B). The content of NO_x_ in culture medium was measured directly by Griess reaction. As shown in Figure 2C, CSD peptides pretreatment decreased the NO_x_ production which was increased by LPS. These data suggested that CSD peptides might have the anti-inflammatory effect on LPS-stimulated AMs.

### Effect of CSD peptides on macrophage phenotype polarization *in vitro*

**Figure 3:**
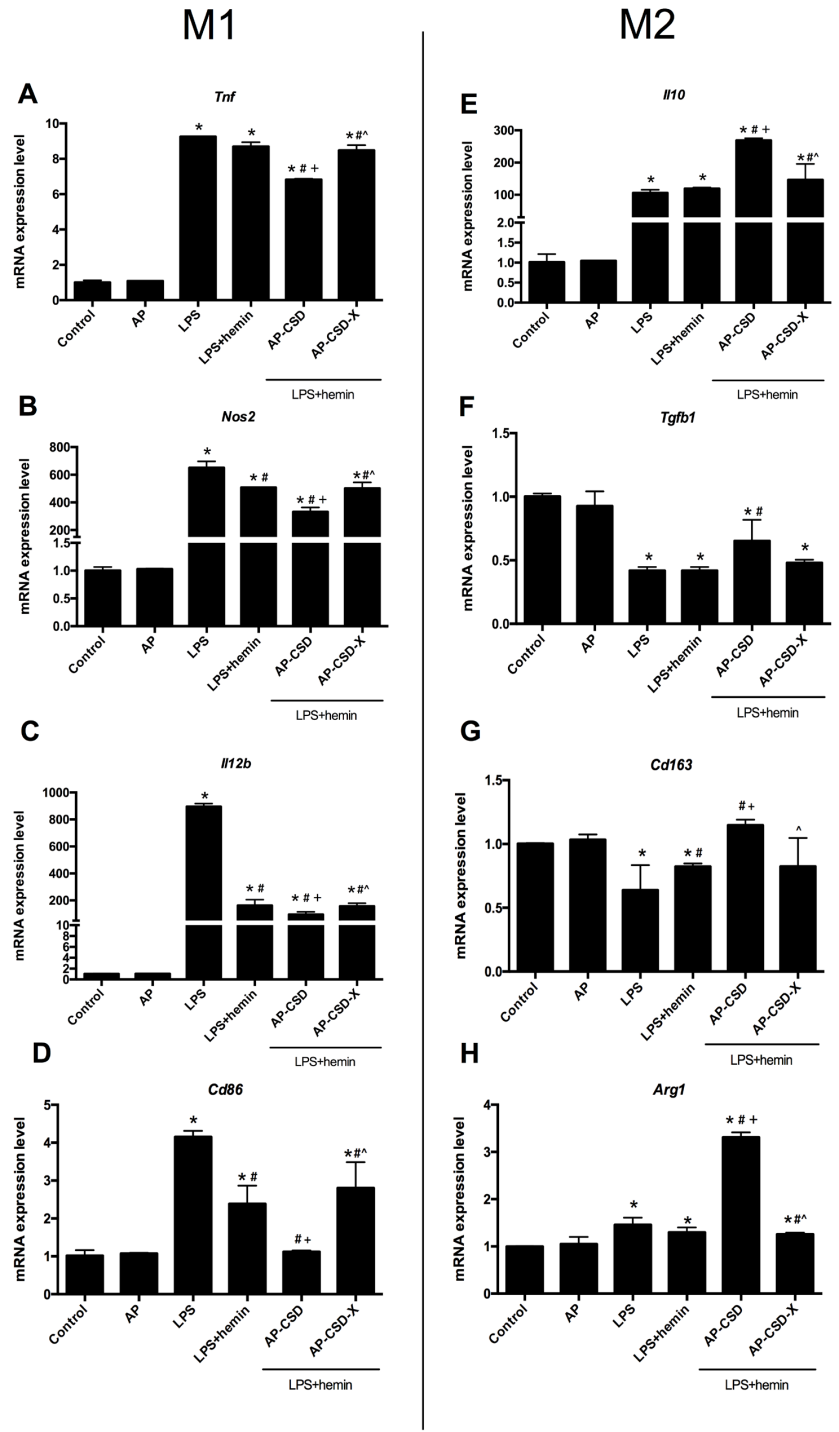
CSD peptides shifted alveolar macrophages polarization toward M2 phenotype **A**.-**D**. The expression of *Tnf*, *Nos2, Il12b* and *Cd86* (the markers of macrophage M1 phenotype), were detected in AMs. **E**.-**H**. The markers of macrophage M2 phenotype such as *Il10*, *Tgfb1*, *Cd163* and *Arg1* expression, were detected in alveolar macrophages. Values represented mean ± SD (*n* = 5 for each group). *, significance compared with control group (*P < 0.05*); #, significance compared with LPS group (*P < 0.05*); +, significance compared with LPS + hemin group (*P < 0.05*); ^, significance compared with LPS + hemin + AP-CSD group (*P < 0.05*).

Because CSD peptides could decrease the expression of pro-inflammatory cytokines in AMs, we supposed that CSD peptides might affect the macrophage polarization. As shown in Figure 3, stimulation of AMs with LPS resulted in increased expression *of Tnf*, *Nos2* and *Il10*, but decreased *Cd206* expression at mRNA levels compared with the control group. Compared with the LPS group and LPS + hemin group, CSD peptides pretreatment down-regulated the mRNA expression of M1 macrophage markers (*Tnf* and *Nos2*), but up-regulated the mRNA expression of M2 macrophage markers expression (*Cd206* and *Il10*).

### Effect of CSD peptides on IκB degeneration in LPS-stimulated AMs

The degradation of IκB proteins is a prerequisite for classical activation of NF-κB. Since the above-mentioned inflammatory cytokines are associated with NF-κB signal pathway, we investigated the effect of CSD peptides on IκB degeneration. As expected, CSD peptides inhibited the degradation of IκB proteins. The results suggested that the anti-inflammatory effect of CSD peptides was at least in partial mediated by the inhibition of NF-κB nuclear translocation.

### Effect of CSD peptides on the co-localization of HO-1/Cav-1 and HO-1 activity in LPS-induced ALI in mice

We investigated whether CSD peptides had anti-inflammatory effect on LPS-induced ALI in mice. The co-localization of HO-1 and Cav-1 in pulmonary tissue was assessed by immunofluorescence. The interaction between HO-1 and Cav-1 were enhanced in LPS group and LPS + hemin group (Figure 5A). CSD peptides pretreatment obviously decreased their co-localization. Additionally, CSD peptides significantly increased HO-1 activity when compared with that of LPS group and LPS + hemin group (Figure 5B). However, zinc protoporphyrin IX (ZnPP), a HO-1 specific activity inhibitor, effectively abolished the effect of CSD peptides on the HO-1 activity.

**Figure 4:**
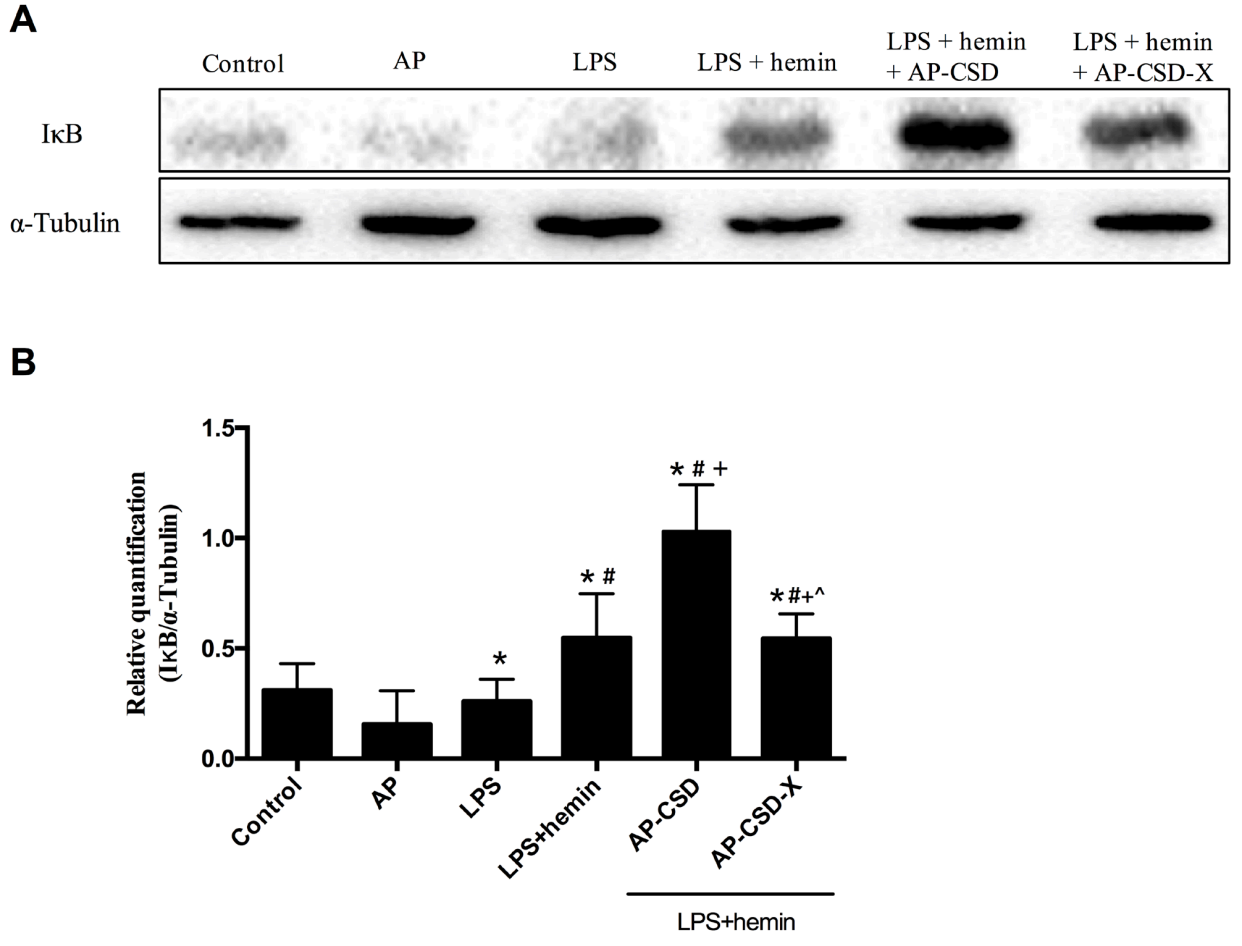
The cytoplasm content of IκB in alveolar macrophages after treating CSD peptides **A**. Representative western blot images of IκB levels in the alveolar macrophages. **B**. Representative analysis to quantify IκB levels. Values represented mean ± SD (*n* = 3 for each group). *, significance compared with control group (*P < 0.05*); #, significance compared with LPS group (*P < 0.05*); +, significance compared with LPS + hemin group (*P < 0.05*); ^, significance compared with LPS + hemin + AP-CSD group (*P < 0.05*).

**Figure: 5:**
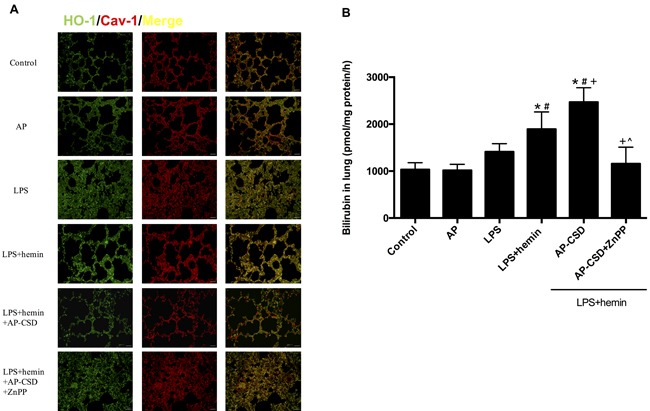
CSD peptides decreased the interaction of HO-1 and Cav-1, and increased the HO-1 activity in LPS-challenged mice **A**. The paraffin sections of lung tissue were immunostained with anti-HO-1 (green) and anti-Cav-1 (red) and analyzed by fluorescence microscopy. The merged images were shown. Scale bar = 20 μm. **B**. HO-1 activity was determined in mice lung tissues. Results were expressed as mean ± SD (*n* = 5 for each group). *, significance compared with control group (*P < 0.05*); #, significance compared with LPS group (*P < 0.05*); +, significance compared with LPS + hemin group (*P < 0.05*); ^, significance compared with LPS + hemin + AP-CSD group.

### Effect of CSD peptides on the pathology changes and biochemical indexes in LPS-induced mice ALI

**Figure 6:**
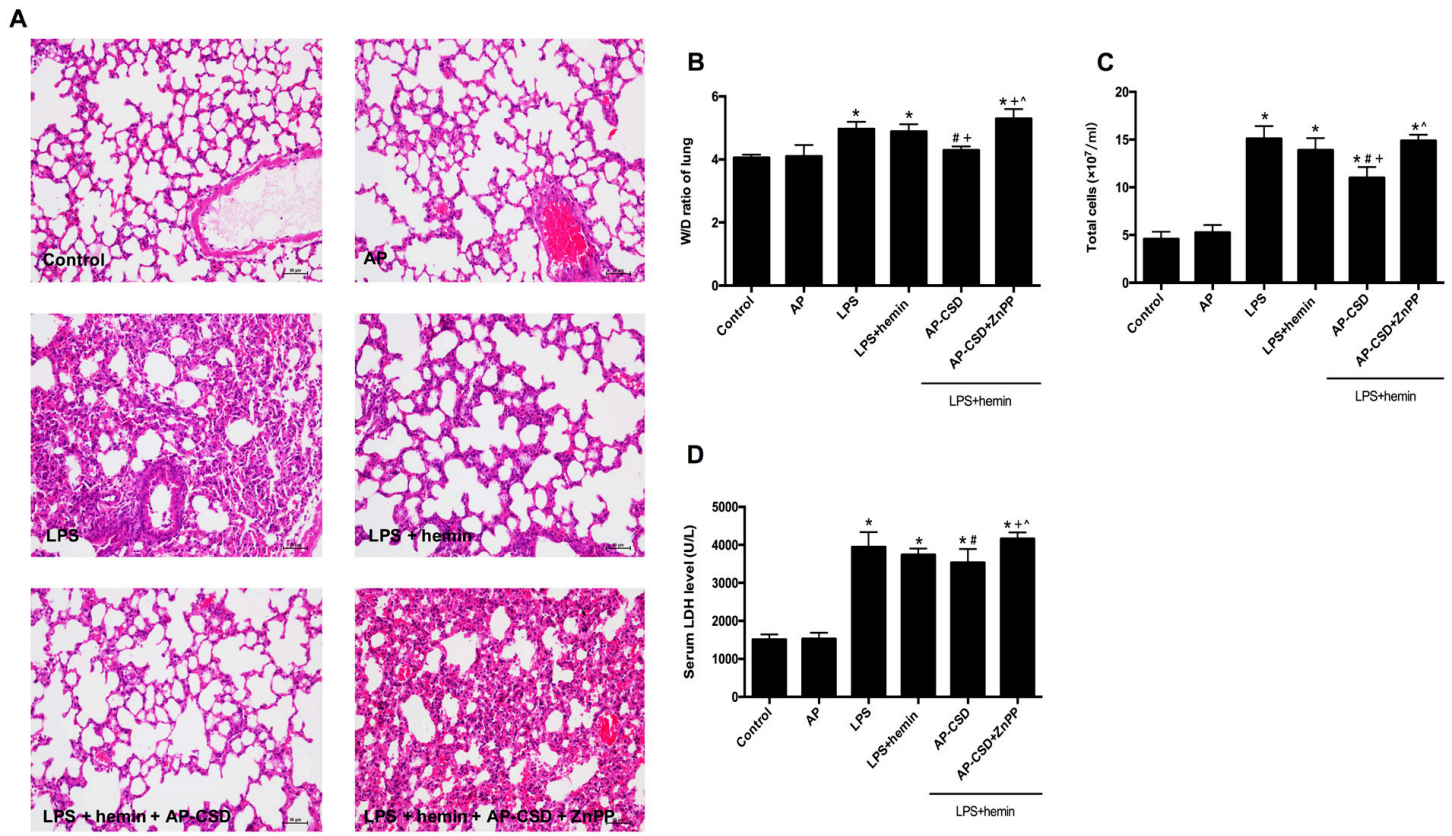
The pathology changes and biochemical indexes in LPS-induced mouse ALI **A**. Hematoxylin and eosin stained lung sections (*n* = 5 each group). Scale bar = 20 μm. **B**. wet/dry weight ratio in lung tissue. **C**. The total cell count in bronchoalveolar lavage fluid. **D**. The serum lactate dehydrogenase activity was determined by a commercial kit. Values represented mean ± SD (*n* = 5 for each group). *, significance compared with control group (*P < 0.05*); #, significance compared with LPS group (*P < 0.05*); +, significance compared with LPS + hemin group (*P < 0.05*); ^, significance compared with LPS + hemin + AP-CSD group (*P < 0.05*).

H&E-stained sections showed that LPS caused interstitial edema, alveolar disarray and neutrophil infiltration in the lung tissue. CSD peptides markedly ameliorated these pathological changes. Furthermore, CSD peptides administration significantly decreased the W/D ratio in the lung tissue, total cells number in bronchoalveolar lavage fluid, and lactate dehydrogenase activity in the serum (Figure 6). However, ZnPP abrogated the protective effect of CSD peptides on LPS-induced ALI in mice.

### Effect of CSD peptides on inflammatory cytokines production in LPS-induced ALI in mice

**Figure 7:**
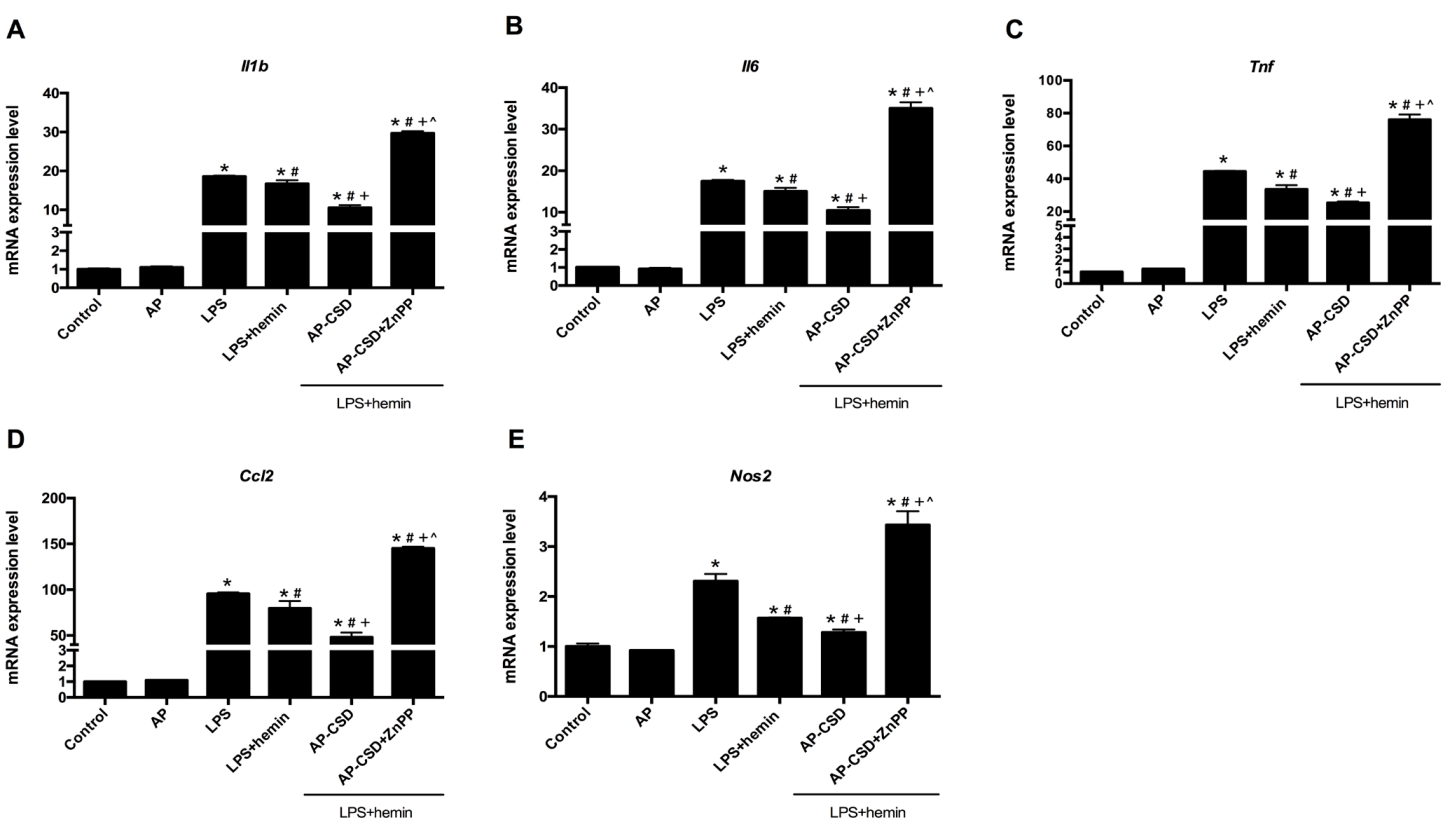
CSD peptides repressed the expression of pro-inflammatory genes in LPS-induced acute lung injury of mice **A**.-**E**. Expression of *Il1b, Il6, Tnf, Ccl2* and *Nos2* was examined by qPCR. Values represented mean ± SD (*n* = 5 for each group). *, significance compared with control group (*P < 0.05*); #, significance compared with LPS group (*P < 0.05*); +, significance compared with LPS + hemin group (*P < 0.05*); ^, significance compared with LPS + hemin + AP-CSD group (*P < 0.05*).

To confirm the anti-inflammatory effect of CSD peptides on LPS-induced ALI in mice, we detected the inflammatory cytokines (*Il1b, Il6, Tnf* and *Ccl2*) and *Nos2* by qPCR. As shown in Figure 7, treatment with LPS could enhance the production of pro-inflammatory cytokines in the lung tissue while CSD peptides decreased these cytokine expressions. However, ZnPP reversed the effect of CSD peptides on the mRNA expression of inflammatory cytokines*.* In summary, these data suggested that the anti-inflammatory effect of CSD peptides on LPS-induced ALI was dependent on HO-1 activity.

## DISCUSSION

This study aimed to look for the ability of CSD peptides to deprive the enzyme activity inhibit of Cav-1 toward HO-1. We artificially synthesized the CSD peptides and investigated their effects on HO-1 activity. As expected, CSD peptides enhanced HO-1 activity and alleviated pulmonary inflammation, which could be supported by the following evidences: (1) CSD peptides decreased the compartmentalization of HO-1 and Cav-1 on plasma membrane, raised HO-1 activity, and down-regulated the expression of pro-inflammatory cytokines in LPS-challenged AMs and mice; (2) CSD peptides drived macrophage polarization from M1 to M2 phenotype *in vitro*, inhibited the IκB degeneration and obviously ameliorated the pathology changes *in vivo*.

A continuous and uncontrolled inflammation in lungs recognized as a significant role in the pathogenesis of ALI induced by sepsis. Therefore, inhibition of inflammatory responses may be a key strategy to attenuate the disease progression of ALI [20]. Data from the present study revealed that LPS-induced pulmonary pathological characteristics and inflammatory cytokines could be markedly attenuated by CSD peptides. Therefore, it is likely that CSD peptides maybe is a potential novel agent to treat ALI induced by sepsis.

Existing knowledge has revealed the diverse contribution of macrophage-derived cytokines to the injury and resolution phases of ALI. With onset of inflammation, alveolar macrophages (M1 phenotype) are thought to be the primary source of pro-inflammatory cytokines such as IL-1β and TNF-α. On the contrary, M2 macrophages participate in the resolution of inflammation and are known to be beneficial in the outcome of several inflammatory diseases. Reprogramming inflammatory macrophages toward an M2 phenotype may be involved in the resolution phase of ALI [21]. HO-1 over-expression can influence macrophage polarization toward M2 phenotype by the transcription factor, nuclear factor erythroid-2 related factor [22, 23]. In the study, we assessed the role of CSD peptides in macrophage polarization and found that CSD peptides droved the phenotypic shift to M2 macrophages, which implied that CSD peptides might be a potential treatment for ALI.

The complex network of inflammatory cytokines and chemokines plays a major role in mediating, amplifying, and perpetuating the lung injury process [24]. Thus, suppression of inflammatory responses may be a potential strategy to attenuate the progression of ALI. Large numbers of evidences have shown the HO-1 induction by natural compounds or gene therapy approaches ameliorated the injury in LPS-induced ALI *via* its anti-inflammatory effects [4, 25–28]. In line with this, we also showed that hemin down-regulated the pro-inflammatory cytokine expression such as IL-1β, IL-6, TNF-α and MCP-1 and iNOS and decreased IκB degradation *in vitro* and *in vivo*. Importantly, CSD peptides enhanced the anti-inflammatory effect of HO-1 inducer. These findings suggested that CSD peptides have a predominantly anti-inflammatory function.

In summary, our study is the first report that CSD peptides ameliorated LPS- induced ALI *via* enhancing HO-1 activity. The study may offer a new agent for treating ALI. Definitely, more rigorous experiments are needed to confirm the potential clinical benefits of CSD peptides.

## MATERIALs AND METHODS

### Animal

BALB/c mice (8-10 weeks, weighted 19-23g) were purchased from SLAC Laboratory Animal Co. Ltd. (Shanghai, China). All animals were allowed to acclimate to the appropriate environment for 7 days. All experiments were performed in accordance with relevant laws and institutional guidelines, and approved by the Ethics Committee for the Use of Experimental Animals in Jiangnan University.

### Peptides

Peptides, corresponding to the full-length CSD peptides (amino acids 82-101; DGIWKASF-TTFTVTKYWFYR, named CSD peptides) and the scrambled control peptide (Cav-X, WGIDKAFFTTSTVTYKWFRY). Peptides were synthesized as a fusion peptide to the C-terminus of the *Antennapedia* (AP) internalization sequence (RQIKIWFQNRRMKWKK) by purified high-performance liquid chromatography and analyzed by mass spectrometry to confirm purity more than 95% by China Peptides Co. Ltd. (Shanghai, China). Desiccated peptides were weighed, and dissolved in DMSO to 10 mM, and then diluted with distilled water to 1 mM.

### Alveolar macrophages culture

Primary alveolar macrophage (AMs) were harvested from BALB/c mice as previously described [29]. Animals were euthanized with an intraperitoneal injection of pentobarbital (100 mg/kg). Intratracheal lavage of the lungs was performed three times with instilling 1-3 ml aliquots of cold normal saline. After centrifugation for 10 min at 1,000 rpm at 4°C, the cell pellet was collected and re-suspended in DMEM containing 10% fetal bovine serum, 100 U/ml penicillin, and 100 μg/ml streptomycin. Cells number and viability were assessed by Trypan blue exclusion assay. Then, cell suspension was inoculated to the plates according to experiment plan. Cells were cultured at 37 °C in a 5% CO_2_ humidified incubator for 2-3h. Non-adherent cells were removed by gentle agitation. Then cells were synchronized culture by serum-free medium for 12 h before each experiment.

### Mouse models of LPS-induced ALI

Thirty-six male mice randomly divided into 6 groups (*n* = 6): Control group, AP peptide group, LPS group, LPS + hemin group, LPS + hemin + CSD group and LPS + hemin + CSD + ZnPP group. Mice in control group were treated intraperitoneally with normal saline; AP group daily treated intraperitoneally with AP peptide (4 mg/kg); LPS group treated intraperitoneally with normal saline and intratracheal instilled LPS (5 mg/kg); LPS + hemin group treated intraperitoneally with hemin (50 μM/kg) and intratrachealy adminstrated LPS(5 mg/kg) after 24 h; LPS + hemin + CSD group treated intraperitoneally with hemin and daily treated intraperitoneally with CSD peptides (4 mg/kg), then intratracheally administrated with LPS after 24 h [30]; LPS + hemin + CSD + ZnPP group treated intratracheally with ZnPP (50 μM/kg) 2 h before adminstration LPS, hemin and CSD peptides. At 24 h after LPS administration, right lungs were collected for subsequent biochemical analysis or determined the W/D ratio. Left lungs were fixed with 4% paraformaldehyde for histological examination. In separate experiments, total cell counts in bronchoalveolar lavage fluid were assessed.

### Nitric oxide content in culture supernatant

1×10^6^ AMs were seeded on six-well cell culture plates, followed by the treatment with 10 μM peptides for 6 h before LPS and hemin stimulation. AMs were treated with LPS (100 ng/ml) and hemin (20 μM) for an additional 16 h. After 16 h of incubation, the concentration of nitric oxide the in culture supernatant was determinated using nitric oxide assay kit (Beyotime, China).

### Quantitative polymerase chain reaction (qPCR)

Total RNA was extracted using the RNAprep Pure Cell/Bacteria Kit or RNAprep Pure Tissue Kit (Tiangen, China). The yield and purity of RNA samples were assessed by the ratio of absorbance at 260 and 280 nm. A volume of 1 μg of total RNA was reversely transcribed to cDNA by PrimeScript RT Master Mix (Takara, Japan) according to the manufacturer's instructions. In brief, qPCR was performed according to the SYBR Premix Ex Taq system protocol (Takara, Japan). All primers in this study were synthesized by Sangon Biotech Co. Ltd. (Shanghai, China) and listed in the Supplemental Material Table 1. Quantification was performed using the 2^-∆ ∆Ct^ method with GAPDH as a reference gene. Expression in the control group was normalized to 1.

### Western blot

AMs or lung tissues were washed thrice with cold phosphate buffer saline (PBS) and homogenized in RIPA buffer with protease and phosphatase inhibitor mixture (Roche Diagnostics) for 30 min on ice. After centrifugation for 10 min at 12,000 rpm at 4°C, the supernatant was collected. 30μg of protein was separated by 10% SDS-PAGE electrophoresis and transferred to nitrocellulose membranes (Millipore, US) and hybridized using standard procedures. The blots were probed with anti-HO-1 (diluted 1: 1,000, Abcam, MA, USA), anti-Cav-1 (diluted 1:1,000, Abcam, MA, USA), anti-IκB(diluted 1:1,000, Cell Signaling Technology, Inc.)and anti-α-Tubulin (diluted 1:1,000, Abcam, MA, USA) antibodies. After incubation with Horseradish Peroxidase- conjugated to IgG secondary antibodies (diluted 1:5000, Boster), the relative signal intensity of bands was determined and standardized by chemiluminescence imaging system ChemiDoc (Bio-Rad) and the Image Lab software (Bio-Rad). Protein relative expression was normalized to α-Tubulin.

### Co-immunoprecipitation assays

AMs were seeded in 10-cm cell plate culture and after stimulated as indicated. For immunoprecipitation technique, we used the protein A/G magnetic beads (Biotool, China). In brief, almost 1× 10^7^ cells were lysed in RIPA buffer supplemented with complete protease inhibitor mixture (Roche Diagnostics) for 30 min on ice, and cells debris were collected by centrifugation at 12,000 rpm for 10 min at 4°C. The supernatant was collected and quantified for protein concentration and adjusted to 1 μg/μl. Then, 5 μg of each immunoprecipitation antibody was added to 500 μg of protein extract. The mixture was incubated with continuous agitation 12 h at 4°C. After 12 h, 35 μg of magnetic beads were added and incubated 3 h in agitation at 4°C; beads were washed 3 times with RIPA buffer. The target antigen was eluted with 1×loading buffer in the final volume of 50μl. 20 μl of target antigen in loading buffer was used and detected by western blot assay. Western blot signals were detected using the ECL chemiluminescence system (Millipore).

### HO-1 activity

Cells and tissues were homogenized in RIPA buffer with protease inhibitor mixture. HO-1 activity was measured by the spectrophotometric determination of bilirubin production as described previously [31]. Final reaction concentrations were: 25 μM hemin, 2U cytochrome P450 reductase, 1 mM β-NADPH, 5mM deferoxaminemesylate salt, 0.25 mg/ml protein, and 2 mg/ml partially purified rat liver biliverdin reductase preparation. Reaction mixtures were incubated for 60 min in a 37 °C water bath in the dark. The reactions were terminated by addition of the same volumes of chloroform. The bilirubin concentration was spectrophotometically determined by measuring the difference in absorbance between 465 and 530 nm, with a molar extinction coefficient of 40/mM/cm.

### Immunofluorescence microscopy

Cells were seeded on 15mm confocal microscopy plate at a density of 1×10^4^ per plate for the incubated time. After rinsed three times with PBS, cells were fixed in 4% paraformaldehyde and transparentized for 20 min in 0.2% Triton X-100. The cells were grown on coverslips and treated for 1 h with various concentrations of curcumin (1, 5 or 10 μM) followed by stimulated with 30 mM glucose for 72 h. The cells were fixed in 4% paraformaldehyde with 0.1% Triton X-100 for 30 min at 4 °C. Anti-cav-1 antibody and anti- HO-1 antibody were used as primary antibodies for 1 hour at 4 °C. FITC- and Texas Red-conjugated IgG antibodies (1:200, Santa Cruz) conjugated was used as secondary antibodies at 37 °C for 1 hour. All sections were observed under immunofluorescent microscopy (LSM 510 confocal laser scanning microscopy, Zeiss).

### Lung histology and biochemical indexes

The left lungs were fixed in 4% paraformaldehyde for 48 h. After paraffin embedding, each sample was sliced into 4 μm sections and stained with hematoxylin and eosin. The severity of lung injury was evaluated by examining interstitial inflammation, inflammatory cell infiltration, and tissue edema using a Nikon microscope (Nikon, Japan). The activity of lactate dehydrogenase (LDH) was determined in the serum by LDH kit (Nanjing Jiancheng, China).

### Statistical analysis

The results were presented as mean ± standard deviation. The one-way analysis of variance (ANOVA) was performed with a SPSS package. Differences were determined to be statistically significant when *P < 0.05* was attained.

## SUPPLEMENTARY MATERIALS TABLE



## Abbreviations

ALI: Acute lung injury
Cav-1: Caveolin-1
CSD: Caveolin-1 scaffolding domain
AP: *Antennapedia*
HO-1: Heme oxygenase-1
AMs: Alveolar macrophages
LPS: Lipopolysaccharide
IL-1β: interleukin-1β
IL-6: interleukin-6
TNF-α: tumor necrosis factor-α
MCP-1: monocyte chemoattractant protein-1
iNOS: inducible Nitric Oxide Synthase
ZnPP: zinc protoporphyrin
NF-κB: nuclear factor-κB
LDH: lactate dehydrogenase
