# Supplementary material for: Caveolin-1 scaffolding domain peptides enhance anti-inflammatory effect of heme oxygenase-1 through interrupting its interact with caveolin-1
Source: Oncotarget. 2017 Mar 29;8(25):40104–14. doi: 10.18632/oncotarget.16676 (PMC5522314; doi:10.18632/oncotarget.16676)
Supplement: Supplementary file 1 [file oncotarget-08-40104-s001.pdf]

Caveolin-1 scaffolding domain peptides enhance anti-inflammatory effect of heme oxygenase-1 through interrupting its interact with caveolin-1

Supplementary Material

Supplementary Table 1: Primers used in this study for qPCR

Protein	Gene	Forward	Reverse
IL-1β	*Il1b*	5'-GAAATGCCACCTTTTGACAGTG-3'	5'-CTGGATGCTCTCATCAGGACA-3'
MCP-1	*Ccl2*	5'-ATCCCAATGAGTAGGCTGGAGAGC-3'	5'-CAGAAGTGCTTGAGGTGGTTGTG-3'
TNF-α	*Tnf*	5'-CCTGTAGCCCACGTCGTAG-3'	5'-GGGAGTAGACAAGGTACAACCC-3'
iNOS	*Nos2*	5'-CATTGGAAGTGAAGCGTTTCG-3'	5'-CAGCTGGGCTGTACAAACCTT-3'
IL-12p40	*Il12b*	5'-AGGCCCAGCAGCAGAATAAATA-3'	5'-GTGCTCCAGGAGTCAGGGTACT-3'
CD86	*Cd86*	5'-AATCCTTTTCTCGGTGTTGG-3'	5'-CTCGGGCTTATGTTTTGAGC-3'
IL-10	*Il10*	5'-TAAGGGTTACTTGGGTTGCC-3'	5'-TTCATGGCCTTGTAGACACC-3'
TGF-β1	*Tgfb1*	5'-GCAACAACGCAATCTATGACA-3'	5'-GAAAGCCCTGTATTCCGTCTC-3'
CD163	*Cd163*	5'- CTGGGATGTCCAACTGCCAT-3'	5'- AATGCTTCCCCCATTCCTGG -3'
Arg-1	*Arg1*	5'-GGCGTTGACCTTGTCTTGTT-3'	5'-CTGTTCGGTTTGCTGTGATG-3'
IL-6	*Il6*	5'-TCTATACCACTTCACAAGTCGGA-3'	5'-GAATTGCCATTGCACAACTCTTT-3'
GAPDH	*Gapdh*	5'-CCTCGTCCCGTAGACAAAATG-3'	5'-TCTCCACTTTGCCACTGCAA-3'
